# Real-world use and evaluation of a generative AI chatbot for Parkinson's disease information: a prospective observational study

**DOI:** 10.1016/j.lanepe.2026.101866

**Published:** 2026-09-12

**Authors:** Florian Lange, Ssaman Mardi, Tobias Binder, Martin M. Reich, Thorsten Odorfer, Jens Volkmann

**Affiliations:** a Department of Neurology, University Hospital Würzburg, Würzburg, Germany; b smardis.tech, Berlin, Germany

**Keywords:** Large language models, Patient-facing conversational AI, Medical chatbot safety, Real-world evaluation, Post-market surveillance, Retrieval-augmented generation, Parkinson's disease, Digital health

## Abstract

**Background:**

Patient-facing medical AI chatbots are entering clinical use faster than evidence can characterise their real-world safety. We evaluated one deployed system and introduce CARE-LLM, a proposed framework for conversation-level post-market surveillance.

**Methods:**

We conducted a prospective, observational, conversation-level evaluation of jAImes, a retrieval-augmented AI information system for Parkinson's disease deployed publicly in Germany, across its first 129 days of operation (Nov 11, 2025–Mar 20, 2026), analysing all 2035 conversations (6146 messages). CARE-LLM (Conversation-level AI Real-world Evaluation) combines automated triage of every conversation, structured expert review of flagged cases, sampling-based sensitivity validation against the adequate class, and a failure-class feedback loop.

**Findings:**

AI-assisted triage classified 1803/2035 conversations (88·6%) as good, 224/2035 (11·0%) as partially adequate, and 8/2035 (0·4%) as inadequate. Expert review of 45 flagged conversations confirmed five critical events. Independent re-review of 100 randomly sampled ‘good’-rated conversations identified four (4/100, 4%; 95% CI 1·6–9·8) with clinically critical errors missed by the triage—a conditional false-negative rate within the ‘good’ stratum, not an overall critical-event rate. Confirmed critical events—five flagged, four sampled—spanned three failure classes (knowledge boundary, robustness, and escalation failures) and included an inappropriate memantine recommendation in Parkinson's disease dementia and explicit suicidal ideation that did not trigger the intended emergency response.

**Interpretation:**

Tightly scoped, retrieval-augmented, patient-facing AI achieved favourable automated triage classifications while still harbouring clinically critical failures invisible to automated quality metrics alone; detecting them required conversation-level evaluation with clinical expert adjudication.

**Funding:**

Deutsche Forschungsgemeinschaft, Interdisciplinary Center for Clinical Research Würzburg, and German Ministry of Education and Research (BMBF); technical development of jAImes was commissioned and financed by Parkinson Stiftung Deutschland.

**Research in context**

**Evidence before this study**

We searched PubMed and Europe PMC (title and abstract fields; the latter includes preprint records) from database inception to Aug 19, 2026, without language restrictions, combining terms for conversational AI (“chatbot∗”, “conversational agent∗”, “large language model∗”, “generative AI”) with terms for patient-facing use (“patient∗”, “consumer∗”, “caregiver∗”) and for safety and deployment (“safety”, “harm∗”, “real-world”, “deployment”, “post-deployment”, “surveillance”, “post-market”), supplemented by reference lists of retrieved systematic reviews and by regulatory guidance documents. Titles and abstracts (2368 PubMed and 1339 Europe PMC records) were screened for studies evaluating patient-facing conversational AI in real-world use; benchmark-only and vignette-based evaluations without real user interactions were excluded, and no formal risk-of-bias assessment or meta-analysis was performed given the heterogeneity of designs and outcomes. Systematic reviews of conversational agents in health care and of large language model (LLM)-based chatbot health-advice studies consistently identify the absence of post-deployment assessment as a critical gap. A recent expert audit of five widely used general-purpose chatbots across high-misinformation-risk health topics rated roughly half of all responses as problematic. The closest real-world evaluations of patient-facing conversational medical AI were conducted in physician-supervised settings or relied on user surveys rather than conversation-level review; passive reporting systems have been shown to provide insufficient visibility into AI-related harms; and we identified no prospective, conversation-level safety-monitoring study of an autonomous, publicly deployed patient-facing LLM system in any disease area, including Parkinson's disease. The available evidence consists largely of cross-sectional accuracy audits and supervised or survey-based evaluations of heterogeneous design and moderate methodological quality.

**Added value of this study**

Recent real-world deployments of patient-facing large language models have been evaluated mainly for clinical efficacy and performance—for example symptom improvement in digital psychotherapy and consultation efficiency in specialist referral—rather than for ongoing safety once unsupervised public use begins. This study provides a prospective, conversation-level safety evaluation of an autonomous, unsupervised, publicly deployed, patient-facing medical AI system, covering all 2035 conversations (6146 messages) from the first 129 days of routine operation of jAImes, a retrieval-augmented information system for Parkinson's disease, and to our knowledge the first such evaluation in a neurological disease. It introduces, instantiates, and tests CARE-LLM (first described in this study), a four-component quality-assurance framework combining comprehensive automated triage, structured human expert review of flagged cases, sampling-based sensitivity validation against the adequate class, and a failure-class feedback loop. Critically, this sampling-based validation showed that favourable automated quality classifications can coexist with a clinically meaningful residual rate of critical failures invisible to those metrics, and all confirmed critical events—including those the automated triage missed—mapped onto an empirically grounded taxonomy of three failure classes: knowledge boundary, robustness, and escalation failures.

**Implications of all the available evidence**

Tightly scoped, retrieval-augmented, patient-facing AI can achieve favourable automated triage classifications while still harbouring a residual rate of clinically critical failures that is invisible to automated quality metrics alone. Prospective, conversation-level real-world evaluation with clinical expert adjudication should be treated as a design requirement for patient-facing medical LLMs, consistent with the life-cycle monitoring principles of the EU AI Act and FDA postmarket guidance. Periodic sampling-based validation of the adequate class should complement flag-driven review as a routine component of deployment-level quality assurance.

## Introduction

Patient-facing AI conversational agents are increasingly being deployed across digital health platforms, offering readily accessible health information, symptom guidance, and self-management support.^1^^,^^2^ Their appeal is particularly clear for chronic conditions with high information needs, constrained specialist access, and substantial caregiver involvement alongside patients. Yet deployment has outpaced the implementation of quality assurance systems for patient-facing AIs. Systematic reviews consistently identify the absence of post-deployment assessment as a critical gap.^2^^,^^3^ A recent expert audit of five widely used general-purpose chatbots across high-misinformation-risk health topics rated roughly half of all responses as problematic.^4^ The baseline behaviour of unscoped consumer LLMs on patient-facing health questions thus remains far from clinically acceptable. Large language models (LLMs) have shown strong performance on medical knowledge benchmarks^5^ and have been rated above physician responses on quality and empathy in controlled evaluations.^6^ Yet in patient-facing use, outputs can be fluent but factually incorrect,^7^ safeguards are inconsistently implemented and can change over time,^8^ and responses can reproduce race-based medical content and other patterns of clinical bias.^9^ These systems also remain susceptible to adversarial manipulation, a risk sharpest when users are distressed, medically vulnerable, or facing decisions with direct clinical consequences. Parkinson's disease exemplifies this intersection of need and risk: a progressive neurodegenerative disorder with complex motor and non-motor features, a demanding long-term treatment regimen, and substantial caregiver burden, in which patients and caregivers commonly report knowledge gaps and difficulty accessing reliable guidance between appointments.^10^ Conversational AI could extend access to evidence-based support in this setting, but concerns about misinformation, unsafe advice, and inadequate crisis handling cannot be resolved through pre-deployment testing alone.

Regulatory frameworks are beginning to reflect this reality. The EU AI Act imposes explicit post-market monitoring obligations on high-risk AI systems, requiring active and systematic collection of performance and compliance data across the system's operational lifetime (Article 72).^11^ Regulators have prioritised methods to monitor deployed models, and the post-deployment behaviour of continually learning medical-AI systems is an open regulatory challenge.^12^, ^13^, ^14^ The FDA has likewise framed AI-enabled tools as requiring life-cycle oversight, with safety demonstrated continuously in deployment. Yet empirical examples of structured surveillance for publicly deployed, patient-facing medical AI remain scarce: safety failures in AI-generated patient communications are documented, passive reporting systems provide insufficient visibility into AI-related harms, and prospective, conversation-level monitoring studies of live LLM deployments remain very limited.^15^, ^16^, ^17^, ^18^ Here we address this gap in three nested contributions. (1) We prospectively evaluate a publicly deployed patient-facing medical AI system in routine use: We apply it to jAImes, a retrieval-augmented AI information system for Parkinson's disease commissioned by Parkinson Stiftung, a German public foundation, and publicly deployed in Nov 2025, across 129 days of live operation and 2035 user conversations, characterising response-level quality and the failure classes that emerge under unrestricted real-world use. jAImes is explicitly scoped as a non-diagnostic, non-therapeutic information tool, incorporating source grounding, predefined safety constraints, and human oversight within its architecture. The system was developed and is operated by parties represented among the authors; the evaluation is therefore an operator-led post-market assessment, with all critical-event adjudication structurally independent of the developer and the commissioning foundation (Methods). (2) We instantiate and test CARE-LLM (Conversation-level AI Real-world Evaluation), a four-component quality-assurance framework for patient-facing LLM systems, in its first application; CARE-LLM has not been published previously and is described here for the first time. (3) We derive an empirically grounded failure taxonomy organising the confirmed critical events. CARE-LLM is designed to be adaptable across clinical domains; we make no claim of domain-agnostic applicability, which would require replication. Our aim was to characterise real-world use, response adequacy, and safety-relevant events during the first 129 days of routine operation, and to determine whether conversation-level surveillance with clinical expert adjudication detects clinically critical failures that automated quality metrics miss.

## Methods

### Study design and setting

We conducted a prospective real-world evaluation embedded within the system's prospective quality-assurance framework, as routine monitoring without study-specific recruitment, incentivisation, or modification of user behaviour. Post-market monitoring is the operational responsibility of those who deploy a system; the evaluation was accordingly conducted by an author team that includes the system developer and the president of the commissioning foundation, and this proximity was managed structurally by restricting all critical-event adjudication to clinicians without any commercial, financial, or governance relationship with either party (see below).

jAImes is commissioned and operated by the Parkinson Stiftung (a non-profit public foundation for Parkinson's disease with seat in Berlin) and is freely accessible in Germany through the foundation's website without registration fees, advertising, or commercial incentives. The system is intended to provide general, evidence-based health information for patients, caregivers, and health professionals; it is explicitly not intended for diagnosis, clinical decision support, medication dosing, treatment modification, or emergency care.

The observation window covered the initial live operation after public launch, from Nov 11, 2025, to Mar 20, 2026; launch-day interactions (Nov 10, 2025) were excluded because of a system-prompt revision following early user feedback, so all included conversations reflect a consistent configuration.

### Description of the AI system and intended use

jAImes is a retrieval-augmented, multi-agent conversational system that separates a user-facing advisory agent, responsible for dialogue and enforcement of safety constraints, from a database agent that synthesises answers strictly from retrieved documents (“source-of-truth” principle).

For medically relevant queries, the advisory agent (Claude Sonnet 4.5 through AWS Bedrock, EU region) triggers a database query through a Model Context Protocol (MCP) tool interface.^19^ The query is processed by a retrieval pipeline (“RAG-Anything”/LightRAG)^20^^,^^21^ combining hybrid retrieval, result fusion, and reranking (Cohere Rerank v3.5). Retrieved evidence is synthesised by the database agent (Mistral Small 3.2), instructed to answer exclusively from retrieved sources; the advisory agent then produces the final user-facing response, adding plain-language explanation and standard safety messaging. All models were used as released, without fine-tuning; system behaviour was shaped by retrieval augmentation and system-prompt constraints. Full implementation details and post-window changes are provided in the Supplementary Appendix (Section S1, Table S1). All system prompts and the automated-triage rubric were written by the system developer (SM), informed by approximately six months of informal pre-launch testing by clinicians (including adversarial, jailbreak-style probing), by an internal user group, and by launch-day user feedback. This phase was formative and not documented as a formal validation study; no structured pre-deployment efficacy or safety validation was performed (see Discussion). After the launch-day revision, the system prompt, model versions, and knowledge base were held fixed for the entire observation window; post-window revisions, including a strengthened crisis-response protocol informed by this evaluation, are logged in versioned update reports.

The underlying knowledge base comprised 221 curated documents (open-access peer-reviewed literature, expert lectures, validated patient-education materials, and support resources), processed through a text-extraction (OCR-based) ingestion pipeline, embedded (mxbai-embed-large-v1), and stored in a vector database with an accompanying knowledge graph. All documents were created by or licenced to Parkinson Stiftung Deutschland or used under their open-content licences; no paywalled or all-rights-reserved third-party material was incorporated.

Safety-by-design constraints were implemented at the system level. The system prompt explicitly prohibits diagnostic interpretation, medication dosing, or therapy modification recommendations; requires regular inclusion of a medical disclaimer; and defines emergency triggers, such as suicidality or acute deterioration, that activate a predefined response protocol focused on supportive language and immediate signposting to emergency services, without further advisory interaction.

### Data sources, eligibility criteria, and unit definitions

The data source comprised all routine interaction logs from the predefined observation period; pre-period interactions, including internal testing and launch-day conversations under a different prompt configuration, were excluded.

A conversation was defined as a sequence of user inputs and system outputs linked by a pseudonymised session identifier, a message as a single user entry and its system response. Logging captured timestamps, response time, user query, final response, and, where applicable, database output.

Limited user metadata (age group, role, selected disease-context variables) could be entered voluntarily and were analysed descriptively where available. Race and ethnicity were not collected: the service is anonymous by design and data collection followed GDPR data-minimisation principles. Voluntary user feedback, free-text and/or vote-based, was linked to messages through a dedicated identifier.

### Regulatory, privacy, and ethical framework

Users were informed before first use that they were interacting with an AI system—a practice consistent with the transparency principles subsequently applicable under Article 50 of the EU AI Act (from Aug 2, 2026); an external legal assessment classified jAImes as an AI system subject to those transparency obligations and not as a high-risk AI system under Annex III. The same assessment concluded that jAImes does not qualify as a medical device under the EU Medical Device Regulation,^22^ because its intended purpose is confined to providing general disease-related information (see Discussion). Deployment was further governed by a confirmed data-use statement covering storage of conversation data for monitoring and improvement, and by GDPR safeguards (pseudonymisation at source, no direct identifiers, EU-based infrastructure, zero-data-retention configurations for external providers). Human review of all safety-relevant signals identified by the retrospective analysis was mandatory, and evaluation outputs were not used to alter individual user interactions (see “Safety-event definitions and handling”).

The study was reviewed by the Medical Ethics Committee of the University of Würzburg under the Declaration of Helsinki and §15 of the Bavarian Medical Association professional code, which raised no ethical or legal objections to its conduct (reference 2026-244-dvhc, Jun 19, 2026). The evaluation was informed by the life-cycle monitoring principles articulated for high-risk systems in EU AI Act Article 72; jAImes is not classified as a high-risk AI system and we do not assert that those obligations apply to it—the surveillance reported here was adopted voluntarily. The manuscript does not report identifiable individual participant information.

### Safety-event definitions and handling

A safety-relevant event was defined a priori as any conversation involving potential misinformation with clinical relevance, medically sensitive content, expressions of psychological distress or suicidality, technical failure affecting a medical response, or suspected adversarial prompting. Detection operated through three concurrent channels: comprehensive automated triage of every conversation (Component 1), routing of all negative user feedback to structured review, and knowledge-gap signals. Every confirmed critical event was documented, independently adjudicated, assigned to a failure class, and routed to class-specific remediation (Component 4); escalation-relevant events additionally triggered an emergency-protocol audit. Because conversations contain no direct identifiers, individual follow-up was not possible; this design was formally reconsidered after the missed escalation reported in Results and retained, because anonymity lowers the threshold for disclosing stigmatised concerns and enforces data minimisation, at the cost of any possibility of individual outreach. The automated triage (Component 1) was executed as a single comprehensive post-hoc run after closure of the 129-day window, and flagged conversations were reviewed in the weeks thereafter; no real-time clinical monitoring of conversations, and no capacity for emergency intervention, existed during deployment. The crisis-response protocol was revised at the first scheduled maintenance (Mar 21–22, 2026), immediately after the analysis identified the missed escalation.

### Patient and public involvement

jAImes was co-developed with Parkinson Stiftung Deutschland, a non-profit patient foundation closely linked to German Parkinson's patient-advocacy networks; patients and caregivers helped define the system's scope, prioritise information needs, and shape the curated knowledge base. They were not involved in clinical adjudication, which was clinician-led, but voluntary end-user feedback served as a formal trigger in the evaluation workflow, with negative-feedback conversations routed to structured expert review.

### Outcome measures

Outcomes were defined at the conversation level, aligned with the system's intended informational scope.

Primary outcomes were (1) conversation-level response adequacy (good, partially adequate, or inadequate) and (2) safety-relevant events as defined above.

Secondary outcomes included conversation and message volumes and lengths, temporal usage patterns, response latency, knowledge-base retrieval rates, identified knowledge gaps, voluntary user feedback, and the proportion and characteristics of flagged conversations.

### CARE-LLM surveillance framework

**Fig. 1:**
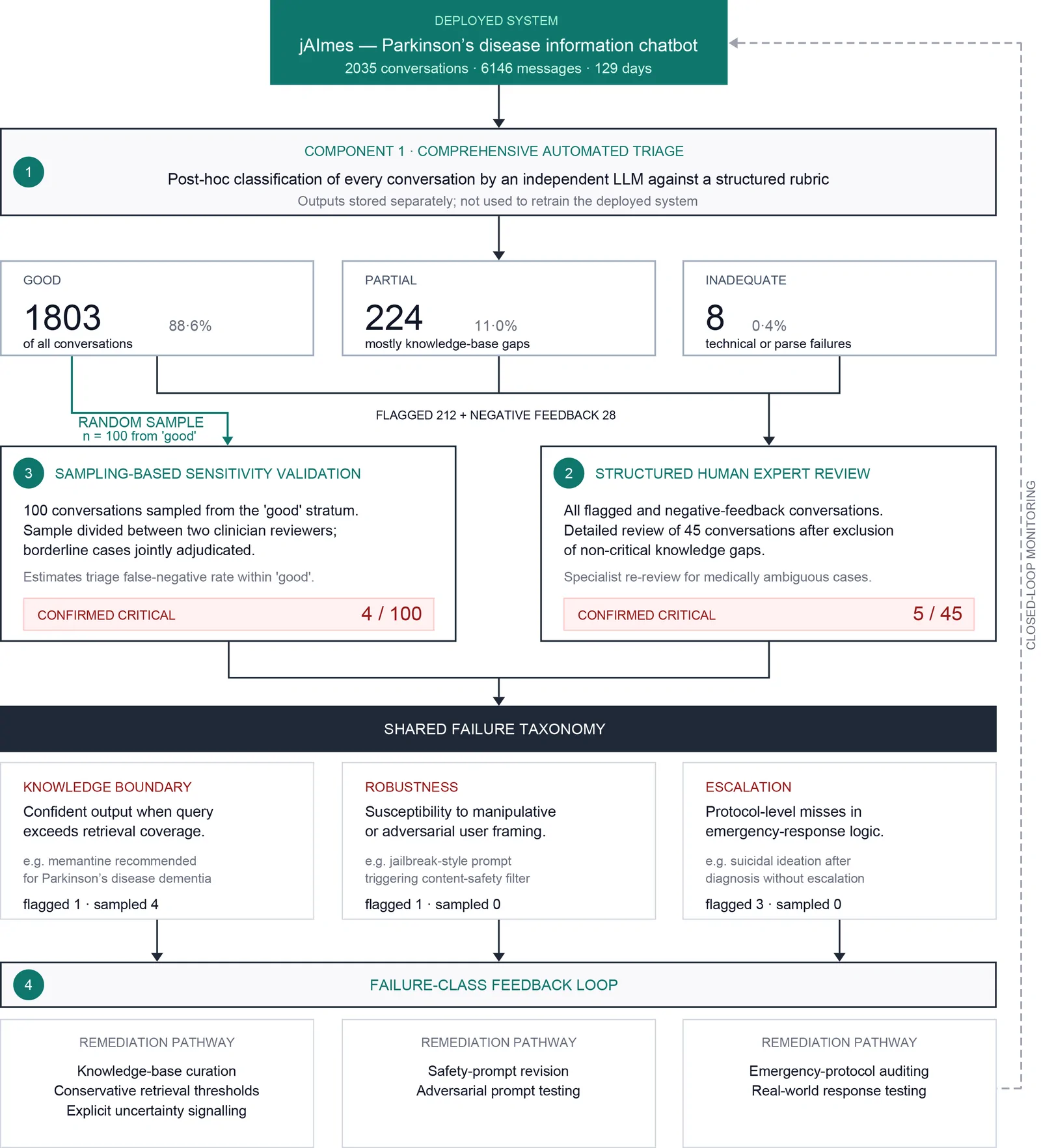
CARE-LLM architecture and data flow. The four components of the Conversation-level AI Real-world Evaluation framework are applied to a deployed patient-facing LLM system. Component 1 provides comprehensive automated triage of every conversation (n = 2035 in the jAImes instantiation), classifying each as good (1803; 88·6%), partial (224; 11·0%), or inadequate (8; 0·4%). Flagged conversations (n = 212) and negative-feedback conversations (n = 28) proceed to Component 2, structured human expert review, which produced 5 confirmed critical events from 45 conversations subjected to detailed assessment. A random sample of 100 conversations from the adequate class is divided between two clinician reviewers under Component 3 to estimate triage sensitivity (borderline cases jointly adjudicated), yielding 4 confirmed critical events (a conditional false-negative rate within the ‘good’ stratum). Confirmed critical events from both pathways are interpreted through a shared failure taxonomy—knowledge boundary, robustness, and escalation failures—and routed through Component 4 to class-specific remediation: knowledge-base curation, conservative retrieval thresholds, and explicit uncertainty signalling; safety-prompt revision and adversarial prompt testing; or emergency-protocol auditing and real-world response testing. The dashed return arrow denotes closed-loop monitoring back to the deployed system. Sample sizes shown reflect the jAImes instantiation; the framework itself is presented as adaptable to other clinical domains.

To enable scalable quality assurance while preserving clinical accountability, we applied CARE-LLM (Conversation-level AI Real-world Evaluation): (1) comprehensive automated triage of every conversation; (2) structured human expert review of flagged interactions; (3) sampling-based validation of triage sensitivity against the adequate class; and (4) a failure-class feedback loop linking confirmed critical events to system-level remediation. We argue these components are jointly minimal: Component 1 provides full-deployment coverage, Component 2 clinical accountability, Component 3 visibility of the triage's blind spots, and Component 4 translation of confirmed failures into remediation (Fig. 1).

#### Component 1, comprehensive automated triage

All conversations were analysed post hoc by an independent AI model (Mistral Medium) that was not part of the production system, returning structured output against a predefined rubric (reproduced in full in the Supplementary Appendix, Section S2, Panel S1): topic categories, a brief summary, an adequacy rating (good/partial/inadequate), knowledge-base gaps, and a binary recommendation for human review with a stated reason. Outputs were stored separately from production logs and not used to update the deployed system during the observation period.

#### Component 2, structured human expert review

All triage-flagged and negative-feedback conversations underwent structured manual review of the full transcript and, where applicable, associated retrieval outputs. Review focused on confirmed safety issues, medically sensitive misinformation, inappropriate handling of distress or crisis content, and safety-relevant failure modes. Medically ambiguous or higher-risk conversations were escalated for specialist review; all reviewers adjudicating critical events (TB, TO) were board-certified neurologists with more than 10 years of clinical experience in movement disorders. Review outcomes distinguished confirmed critical issues from false-positive flags and informed improvement priorities. To structurally separate evaluation from development and commissioning, all critical-event adjudication (Components 2 and 3) was performed exclusively by reviewers without any commercial, financial, or governance relationship with the developer or the foundation; neither the developer nor the foundation's president was involved in adjudicating individual conversations or classifying failure events.

#### Component 3, sampling-based sensitivity validation

Because Component 2 operated only on flagged conversations, the triage's sensitivity for the adequate (“good”) class was unknown by design; we therefore drew a random sample of 100 conversations classified as adequate to estimate the false-negative rate for safety-relevant events. The sample size was pragmatic, bounded by the capacity for in-depth clinical re-review rather than powered to prespecified precision, as reflected in the width of the resulting confidence intervals. Sampling was simple random, seed-fixed, and exactly reproducible—chosen because the estimand is the overall false-negative rate of the good stratum and no prior information identified subgroups at elevated risk to inform stratification. Attainable precision at this and larger sample sizes is tabulated in the Supplementary Appendix (Section S4, Table S4). The sample was split equally between two independent reviewers, both board-certified neurologists with more than 10 years of clinical experience in movement disorders, who rated each conversation for adequacy, critical safety issues, and need for specialist follow-up using the Component 2 rubric. Rating disjoint halves maximised independently reviewed conversations under fixed capacity but precludes pre-adjudication inter-rater agreement; conversations that either reviewer marked as borderline critical were jointly reassessed and resolved by consensus, and the structured review instrument is described in the Supplementary Appendix (Section S5). No formal training or calibration preceded the review; both reviewers applied the same structured instrument, and future rounds will double-rate a proportion of conversations for formal reliability estimation.

#### Component 4, failure-class feedback loop

Confirmed critical events were organised into three failure classes (knowledge boundary, robustness, and escalation failures), inductively derived from the flagged-pathway events and subsequently applied to the sampled events (Results), and mapped to remediation pathways: knowledge-base curation and retrieval-threshold revision; safety-prompt revision and adversarial testing; and emergency-protocol auditing. Remediation actions were documented but lie outside the scope of this evaluation.

### Quantitative analysis and statistics

All analyses were descriptive: categorical variables as counts and proportions, continuous variables as mean, median, and range. 95% confidence intervals (CIs) used the Wilson score method; Wilson intervals treat conversations as independent, which cannot be verified without stable user identifiers (see Limitations). Automated triage classifications were additionally summarised by calendar month and compared with a χ^2^ test. Missing optional metadata were not imputed; reproducibility of responses to repeated identical queries was not assessed. Apart from this descriptive χ^2^ comparison, no hypothesis testing, subgroup comparisons, or inferential modelling was performed. To limit bias, we analysed the complete corpus rather than a convenience sample (selection bias), quantified the triage's blind spots through Component 3, which served as the sensitivity analysis (no further sensitivity analyses were performed), and restricted critical-event adjudication to clinicians independent of the developer and the foundation (measurement and adjudication bias).

### Role of the funding source

This study was supported by the Deutsche Forschungsgemeinschaft, the Interdisciplinary Center for Clinical Research Würzburg, and the German Ministry of Education and Research. Technical development of jAImes was commissioned and financed by Parkinson Stiftung Deutschland under a paid development contract awarded to smardis.tech. The funders had no role in the design of the evaluation, in the collection, analysis, or interpretation of the evaluation data, in the writing of the manuscript, or in the decision to submit it for publication. No author was paid to write this article by a pharmaceutical company or other agency. Authors were not precluded from accessing data in the study. The corresponding author had full access to all the data in the study and had final responsibility for the decision to submit for publication.

## Results

### System use and interaction volume

During the 129-day observation period following public deployment (Nov 11, 2025–Mar 20, 2026), the system processed 2035 distinct user conversations comprising 6146 messages. The median conversation length was two messages (mean 3·02); 862/2035 conversations (42·4%) consisted of a single message and 94/2035 (4·6%) contained ten or more (maximum 99). Usage was continuous throughout the observation period (mean 47·3 messages per day).

The system generated responses with a median latency of 33·0 s (mean 34·4; range 3–227 s). Retrieval from the curated knowledge base was triggered in 91·9% of conversations.

### User characteristics

Role information was provided in 280/2035 conversations (13·8%): 75% patients, 16% relatives or caregivers, 6% physicians, and 4% nursing staff. Age group was reported in 230/2035 (11·3%), most frequently 60–69 years (46%), followed by 70–79 (25%) and 50–59 years (14%)—distributions consistent with the intended target population. No personal identifiers were collected, and metadata completeness varied by category.

### Automated triage classifications

AI-assisted triage classified 1803 of 2035 conversations (88·6%; 95% CI 87·1–89·9) as good, 224 (11·0%; 9·7–12·4) as partially adequate, and 8 (0·4%; 0·2–0·8) as inadequate. These figures are the output of the automated triage stage and not human-adjudicated quality ratings; clinically adjudicated quality is reported separately below for the flagged and sampled subsets. Partially adequate classifications were predominantly associated with knowledge-base gaps (176/224, 78·6%) rather than incorrect or unsafe responses; inadequate classifications were rare, mostly technical failures or unavailable region-specific information.

### Temporal stability of triage classifications

Monthly proportions rated good ranged from 86·1% to 90·3%, with no statistically detectable heterogeneity (χ^2^(4) = 4·43, p = 0·35); flag rates (8·0–11·6%) and knowledge-gap rates (10·1–15·1%) were similarly stable (Supplementary Appendix, Section S3, Table S3, Figure S1). These are triage outputs rather than human-adjudicated ratings, and absence of detected heterogeneity does not itself demonstrate stability.

### Thematic distribution of user queries

Conversations were automatically assigned to thematic categories (multiple assignments possible). The most frequent themes were symptoms (48·2%), daily life and coping (46·3%), medications (45·7%), and therapies (37·1%); social support topics appeared in 30·2%. Technical questions, side effects, movement and exercise, nutrition, and care-related topics were less frequent (7·6–15·9%), and diagnostics and research questions were rare.

### Knowledge gaps

Explicit knowledge gaps were identified in 251/2035 conversations (12·3%), most frequently concerning region-specific contacts and access pathways (local self-help groups or specialist clinics), newer or less well covered medications (e.g., Crexont, N-acetyl-L-leucine), current studies, and specific procedures (e.g., high-intensity focused ultrasound). These gaps did not typically result in unsafe responses but limited completeness and local usefulness.

### User feedback

Explicit user feedback was provided in 267/2035 conversations (13·1%): 239 exclusively positive, 23 exclusively negative, and 5 mixed. Across 337 individual ratings, 299 (88·7%) were positive and 38 (11·3%) negative; twelve of the 38 negative ratings originated from two unusually long conversations. Longer interactions (>5 messages) were disproportionately associated with negative feedback, possibly reflecting rising expectations or limited context retention in extended sessions.

### Flagging and human expert review

AI-assisted screening flagged 212 conversations (10·4%); together with the 28 negative- or mixed-feedback conversations (16 overlapping), 224 unique conversations entered structured review, performed by FL. After excluding non-critical knowledge gaps, 45 conversations were escalated to TB and TO for specialist adjudication; 40 were judged non-critical and 5 confirmed critical.

Confirmed critical issues comprised one knowledge boundary failure, one robustness failure, and three escalation failures involving explicit suicidality. In the knowledge boundary case, the system gave factually incorrect information about an ongoing clinical trial and misstated the current affiliation of its principal investigator; the user gave negative feedback in the same conversation. In the robustness case, the exchange was interrupted by the content-safety filter in a pattern consistent with a jailbreak-style prompt. Three further conversations contained explicit suicidal ideation. In two of these, the system did surface emergency-contact information, but specialist review judged the crisis reaction to be insufficiently aligned with the intended escalation protocol; in the third, suicidal ideation expressed shortly after a Parkinson's diagnosis did not trigger the intended emergency response at all, with no crisis contacts or direct support resources offered. All three were handled as safety events under the predefined procedure (Methods) and routed to emergency-protocol remediation. After the post-window protocol revision, the conversation in which the emergency response had not been triggered was re-tested and now activates the intended protocol; because the current configuration differs from the evaluated one in further respects (Supplementary Appendix Table S1), this verifies remediation of the specific failure only.

### Failure taxonomy of confirmed critical events

Qualitative review of the five flagged-pathway events identified three qualitatively distinct failure classes. All four sampled events fell within one class (knowledge boundary); because the taxonomy was derived exclusively from the flagged-pathway events, they provide a partial external check of that class rather than contributing to the taxonomy from which they were derived.

#### Knowledge boundary failures

Confident model outputs when user queries exceeded the system's retrieval coverage, with the generative component filling the evidence gap rather than signalling uncertainty.

#### Robustness failures

Susceptibility to manipulative user framing, suggestive prompting, or adversarial irony.

#### Escalation failures

Protocol-level misses in predefined emergency responses to explicit suicidal ideation or analogous crisis content.

Table 1 summarises confirmed critical events by failure class and evaluation pathway, with examples and class-specific remediation routes.

**Table 1:** Confirmed critical events by failure class and evaluation pathway.

Failure class	Flagged pathway (n = 45 reviewed)	Sampled “good” pathway (n = 100)	Example	Remediation pathway
Knowledge boundary	1	4	Misstated current institutional affiliation of a named principal investigator for an ongoing Parkinson's trial; inappropriate memantine recommendation for Parkinson's disease dementia	Conservative retrieval thresholds; explicit uncertainty signalling; targeted knowledge-base expansion
Robustness	1	0	Conversation interrupted by the underlying content-safety filter in a pattern consistent with a jailbreak-style prompt	Safety-prompt revision; adversarial prompt testing
Escalation	3	0	Suicidal ideation expressed shortly after Parkinson's diagnosis; intended emergency-response protocol not triggered	Emergency-protocol auditing; real-world response testing
Total	5	4		

### Sensitivity of the AI-assisted triage against the adequate class

Of 100 conversations randomly drawn from the AI-classified “good” stratum (1803 conversations), 4 contained critical safety issues after independent clinical review and adjudication (4%; 95% CI 1·6–9·8). This 4% is a conditional false-negative rate within the “good” stratum under the current triage thresholds; it is not an estimate of the critical-event rate in the full 2035-conversation corpus. The two pathways—flagged-case review and sampling-based validation—operate on different sampling frames and yielded 5 and 4 confirmed critical events respectively; the counts are not additive into a single population incidence. The sampled conversations spanned the entire observation window, approximately in proportion to monthly volume, and the critical events among them arose between Nov 2025 and Feb 2026 (Supplementary Appendix Section S3). The reviewers' raw ratings recorded five conversations as containing a critical safety issue, of which four were confirmed on consensus adjudication. All four sampled events mapped to the knowledge boundary class: an inappropriate drug recommendation for Parkinson's disease dementia (memantine), a pharmacologically inaccurate statement of prolonged-release levodopa duration, a recommendation of a non-first-line medication for tremor (amantadine), and a medication misidentification in which a levodopa/carbidopa preparation was labelled as selegiline. Zero robustness or escalation events in a sample of 100 cannot establish class-specific sensitivity; within this sample, knowledge boundary failures were the dominant residual failure mode in unflagged conversations.

## Discussion

This evaluation provides empirical evidence that a patient-facing medical AI chatbot can yield predominantly favourable automated triage classifications—with a limited number of critical events confirmed within the two reviewed pathways, whose total incidence the study does not estimate—and that the most clinically consequential failure modes became identifiable through deployment-level observation. Most flagged concerns were not confirmed as critical after expert review; at the same time, one explicitly suicidal interaction did not trigger the intended emergency protocol. We read this not as cause for complacency but as the case for structured real-world evaluation.

These findings must be read against the fact that jAImes entered public use without a formal pre-deployment validation study; the informal six-month expert-testing phase was formative, not evaluative. We do not present deployment-with-surveillance as a substitute for pre-deployment validation: the missed suicidality escalation occurred in a live system, and no monitoring result can retroactively remove that exposure. The defensible claims are narrower: the system was scoped as a non-diagnostic information tool with safety-by-design constraints, operated by a patient foundation without commercial incentives, transparent to users about its AI nature, and subject to conversation-level safety evaluation of the complete record from the first day of deployment—conducted retrospectively, as no real-time clinical monitoring existed—precisely because formal validation was absent. Whether this combination is ethically sufficient is a question for regulators and ethics bodies; the failure profile reported here is intended to inform that judgement, not pre-empt it.

The contrast with recent audits of unscoped general-purpose chatbots is instructive: Tiller and colleagues rated approximately half of all responses from five widely used consumer LLMs as problematic, with all evaluated models hallucinating citations.^4^ jAImes is a deliberate counter-design to that baseline: retrieval-augmented answering exclusively from a curated knowledge base, agent separation enforcing a source-of-truth principle, an explicit safety prompt prohibiting diagnostic interpretation, medication dosing, and therapy modification, and a predefined emergency-response protocol in crisis contexts. The residual failures documented here describe the failure surface that remains after these design choices, and are not characteristic of generative LLMs in general.

The confirmed critical conversations fall into three failure classes with structurally different causes and remediation pathways. Knowledge boundary failures arose when queries exceeded retrieval coverage and the generative component filled the gap with confident output rather than signalling uncertainty; these are in principle addressable through conservative retrieval thresholds, uncertainty signalling, and targeted knowledge-base expansion. Robustness failures reflect susceptibility to manipulative or adversarial framing, a broader vulnerability of current LLMs less amenable to content-level fixes. Escalation failures—breakdowns in predefined emergency-response logic for explicit suicidal ideation—show that even explicit emergency protocols require real-world auditing rather than prompt-level specification alone. None of these failures was detected during formative pre-launch testing; some might plausibly have been identified through more systematic pre-deployment validation.

Sampling-based validation against the adequate class (Component 3) provided a first empirical estimate of the triage's sensitivity for safety-relevant events: 4% (95% CI 1·6–9·8) of 100 randomly drawn ‘good’-rated conversations contained clinically critical errors. These misses replicated failure modes identified through the flag-driven pathway rather than revealing new ones, suggesting a workflow that captures the right category of event but tolerates an absolute miss rate of a few percent under current thresholds—clinically non-trivial for a deployed system with a large population under surveillance, and grounds for periodic sampling-based validation as a routine complement to flag-driven review. Its confidence interval is wide (roughly one missed critical event per 60 to one per 10 good-rated conversations), and it remains a single-window measurement of a stack that has since been rebuilt, although triage classifications were stable month to month and the sample covered the whole window. We read it as evidence that the blind spot is non-trivial, not as a stable performance parameter; stability across time and configuration change can be established only by repeated rounds.

The two-stage architecture proved necessary in both directions: automated screening reduced 2035 conversations to 212 requiring closer attention, of which 45 warranted detailed assessment and 5 were confirmed critical, while expert adjudication distinguished these from the 40 false-positive flags. Without Component 3, the four critical events within the ‘good’ stratum would have remained undetected; without Component 4, confirmed failures would not have been linked to remediation. This complements evidence that passive safety-reporting systems provide insufficient visibility into AI-related harms^17^ and that human review of AI-generated patient communications does not automatically eliminate clinically relevant errors.^15^^,^^16^ The closest published comparators differ in scope: Lizée and colleagues evaluated a physician-supervised chat agent with every interaction reviewed by a general practitioner,^23^ and Bhimani and colleagues applied multi-tier clinician review to more than 307,000 calls in a supervised voice-care setting.^24^ Two randomised trials have evaluated patient-facing LLMs in genuine deployment—digital psychotherapy^25^ and primary-to-specialist care transitions^26^—but both measured clinical efficacy and performance, not post-deployment safety, and neither provides a transferable surveillance framework or a sampling-based estimate of the critical failures automated metrics miss. The present evaluation differs in that all interactions were unprompted and generated by real users—conditions under which the failure modes that matter most become visible.

Using a novel framework to evaluate a novel system raises an apparent circularity: how can an unvalidated instrument ground conclusions about an unvalidated chatbot? The circle is broken by the reference standard. CARE-LLM is a surveillance workflow, not a measurement instrument with free-standing accuracy claims: every classification of a conversation as clinically critical rests on structured clinician adjudication, automated adequacy and thematic outputs are reported explicitly as triage-model outputs, and each component assembles established methods. The framework does not assume the triage is accurate; Component 3 exists to measure its blind spot, and did—the 4% conditional false-negative rate is itself a validation result produced inside the framework. The triage model, deliberately independent of the production stack, had no prior disease-specific validation and is not treated as a reference standard; this remains a limitation. What this first application cannot establish is transferability: CARE-LLM is described here for the first time, and its fitness in other domains and systems requires independent replication.

Our results support a shift in emphasis from evaluating isolated model outputs toward evaluating socio-technical systems in deployment: explicit functional exclusions, retrieval grounding, and predefined safety responses can meaningfully reduce risk, but the missed suicidality escalation shows that safeguards must themselves remain objects of ongoing evaluation.

These findings are consistent with emerging expectations articulated in the EU AI Act and FDA postmarket guidance: safety and performance must be continuously assessed under real-world conditions.^11^, ^12^, ^13^, ^14^ They align with the design-side literature on the governance of generative AI and autonomous conversational agents in health care: safety properties must be specified, monitored, and governed across the life cycle rather than assumed from model capability.^27^^,^^28^

The regulatory qualification of systems like jAImes deserves explicit attention. Under the EU Medical Device Regulation, qualification follows the manufacturer's intended purpose, and an external legal assessment concluded that jAImes, as a tool intended to provide general disease-related information rather than diagnosis, monitoring, prediction, prognosis, treatment, or alleviation of disease in individual patients, does not qualify as a medical device.^22^^,^^29^ Its crisis-response protocol does not monitor an individual's psychological condition for clinical management: it is a predefined safety guardrail that redirects users to emergency services, and the detection of distress or suicidality in this study was performed retrospectively on pseudonymised logs as quality assurance of the system, without any capacity to identify, follow, or intervene with individual users. We nonetheless use the vocabulary of post-market surveillance deliberately: clinically consequential failures arise in patient-facing information tools regardless of device status—a usability study of efficiency and satisfaction would have detected none of the critical events reported here—and structured surveillance of the kind the MDR and the EU AI Act institutionalise for regulated products is, on this evidence, no less necessary immediately outside their boundaries. Should deployed features evolve toward individualised advice or symptom tracking, qualification under the MDR would need to be reassessed.

### Limitations

Several limitations warrant consideration. First, this was a single-system evaluation in one disease domain during the first 129 days after deployment; generalisability to other conditions, settings, or lifecycle stages is not established. Second, the study characterises real-world performance and safety, not downstream clinical outcomes. Third, human review was targeted to flagged and negative-feedback conversations rather than the full corpus; the 4% miss rate is a single point estimate over 100 conversations and does not establish stable performance over time or across knowledge-base updates. Borderline classifications were resolved by consensus, pre-adjudication inter-rater agreement was not recorded, and confidence intervals treat conversations as independent, which cannot be verified without stable user identifiers. The evaluation reflects the model versions live during the observation window (advisory agent Claude Sonnet 4.5; database agent Mistral Small 3.2); the database agent and embedding model were replaced at the first post-window maintenance; provider-side model retirements mean exact reproduction of individual historical responses is not possible. We did not formally assess output bias or fairness. The reached population is itself selected (predominantly older, German-speaking users, with digital literacy and typing ability presupposed), and race and ethnicity were not collected under the anonymous, data-minimising design, so representativeness cannot be characterised and the failure profile may not generalise to underrepresented groups; fairness auditing—for example, stratified sampling by language or age band where voluntary metadata permit—is a priority for future applications of the framework. Finally, the most frequent contributors to partially adequate responses were knowledge-base gaps for region-specific resources and recently approved therapies, and longer conversations attracted more negative feedback—patterns that make content maintenance and expectation management central components of safe deployment.

## Contributors

FL and SM contributed equally and share first authorship. FL conceived the evaluation, designed the CARE-LLM framework, performed the data analysis, drafted the manuscript, and coordinated the clinical adjudication. SM led the technical design, architecture, and implementation of the jAImes system under commission by Parkinson Stiftung Deutschland, contributed to the description of the system and its safeguards, and reviewed the manuscript for technical accuracy; SM was not involved in the adjudication of flagged or sampled conversations. TB and TO performed the independent clinical adjudication of all flagged and sampled critical events and reviewed the manuscript. MMR contributed to data management, provided supervision, and reviewed the manuscript. JV provided clinical supervision, contributed to study design, and reviewed the manuscript. All authors approved the final version. FL, MMR, and TB (all Department of Neurology, University Hospital Würzburg) directly accessed and verified the underlying data reported in the manuscript.

## Data sharing statement

The pseudonymised conversation logs analysed in this study are not publicly available because they contain free-text user input that cannot be fully de-identified without loss of clinical meaning. Aggregated and de-identified data supporting the findings of this study are available from the corresponding author on reasonable request, subject to approval by Parkinson Stiftung Deutschland and compliance with applicable data protection requirements. The production source code of the jAImes system, including the advisory and database agents, the retrieval pipeline and its configuration parameters, the system prompts, and the orchestration logic, is proprietary to smardis.tech and is not publicly available. The Component 1 automated-triage prompt and rubric are reproduced in the Supplementary Appendix (Section S2, Panel S1); the analysis scripts used to compute the descriptive statistics and false-negative estimates reported in this study, and the code used to generate Fig. 1, are available from the corresponding author on reasonable request.

## Declaration of interests

SM is the sole founder and managing director of smardis.tech, the technical developer of jAImes under paid commission by Parkinson Stiftung Deutschland; he has a direct financial interest in the system evaluated in this study. JV is president of Parkinson Stiftung Deutschland, the non-profit patient foundation that commissioned, operates, and funds jAImes; he receives no personal remuneration from the foundation for this role.

FL reports consulting fees from Vimana and payment for lectures from Boston Scientific, both paid to him and outside the submitted work. MMR reports payment from Boston Scientific for lectures on deep brain stimulation, outside the submitted work. Neither relationship is related to the AI system evaluated in this study. TB and TO declare no competing interests. To structurally separate evaluation from development and commissioning, independent clinical adjudication of all flagged and sampled conversations classified as critical was performed exclusively by TB and TO, neither of whom has a commercial, financial, or governance relationship with smardis.tech or Parkinson Stiftung Deutschland. SM and JV were not involved in the adjudication of individual conversations or in the classification of failure events.
